# Future Work Self Salience and Future Time Perspective as Serial Mediators Between Proactive Personality and Career Adaptability

**DOI:** 10.3389/fpsyg.2022.824198

**Published:** 2022-04-27

**Authors:** Hairong Ling, Shanjie Teng, Xuejun Liu, Jing Wu, Xueying Gu

**Affiliations:** ^1^School of Education Science, Nanjing Normal University, Nanjing, China; ^2^Department of Basic Course, Communication University of China, Nanjing, China; ^3^School of Psychology, Nanjing Normal University, Nanjing, China

**Keywords:** proactive personality, future work self salience, future time perspective, career adaptability, college students

## Abstract

In recent years, employment has become a growing problem for Chinese college students, who often face issues of slow employment and lazy employment. Guided by the framework of career construction theory, we explored how proactive personality strengthens career adaptability. A total of 423 Chinese college students effectively completed the online survey. The results showed a positive correlation between proactive personality, future work self salience, future time perspective, and career adaptability. Additionally, proactive personality can directly affect career adaptability through three indirect paths: the separate intermediary effect of future work self salience, future time perspective, and the continuous mediating role of future work self salience and future time perspective. The results indicate that proactive personality increases career adaptability through the mediating role of future work self salience and future time perspective. This study contributes to our understanding of the mechanisms underlying the relationship between proactive personality and career adaptability. Additionally, the findings have implications for the career development of college students.

## Introduction

Opportunities for stable and safe employment have been increasingly diminished by flexible and short-term employment (Spurk and Straub, 2020), which has complicated career development. With the recent economic transformation and popularization of higher education, Chinese college students have increasingly faced employment problems such as slow employment and lazy employment. Because of this, the task of figuring out how to improve the vocational adaptability of college students has become urgent. On the other hand, schooling and preparation for adult work have gradually extended, with people in their 20s and beyond still engaging in these activities (McAdams and Olson, 2009). College students (around 18–25 years old) who are in the period between adolescence and adulthood (i.e., emerging adulthood) do not immediately establish long-term adult roles. Instead, they frequently engage in different experiences and gradually decide their long-term career path by exploring and discovering different possibilities (Arnett, 2000, 2007). Given this context, more researchers have paid attention to proactive factors, future orientation factors, and ability factors that lead individuals to actively construct their careers, such as proactive personality, future work self salience, future time perspective, and career adaptability in individual career development.

Among these factors, career adaptability is considered to be the key to successful career preparation. Career adaptability is a new concept that is developed based on career maturity (Super and Knasel, 1981), which proved a response to the insufficiency of the career maturity construct, apropos adolescent vocational development, to explain adult career-stage development. Savickas (1997) defined it as the core ability of individuals to cope with various challenges brought on by changes in work or roles. In career construction theory, career adaptability is defined by four global dimensions: concern, control, curiosity, and confidence (Savickas, 2012). Among these, career concern prompts the establishment of possible futures, curiosity fuels the exploration of possible selves and occupations, career control affords ownership of a chosen future, and confidence empowers individuals to construct a preferred future and overcome obstacles (Savickas, 2002). These functions entails influence the strategies individuals use to solve problems and the behaviors they enact to align their vocational self-concepts with work roles over the life course. In research, career adaptability is associated with a lot of outcomes, including smoother school-to-work transitions, heightened job search strategies, and greater job satisfaction (Koen et al., 2012). Research by Magnano et al. (2021) shows that college students with high career adaptability are better equipped to explore, plan, and make decisions during career transitions and are more likely to achieve career success. Career adaptability interventions have shown to advance higher employment quality (Koen et al., 2012), greater career success, higher job satisfaction (De Guzman and Choi, 2013), and increased future orientation (Soresi et al., 2012). Educational institutions across the globe have realized that college is a preparatory period for the development of individual careers and that it is very important to help college students develop good career adaptability. Results support that career adaptability resources may be developed through the effective training intervention (Green et al., 2020). Student affairs practitioners in collaboration with instructional design specialists may develop and implement training interventions that further students’ career adaptability in tandem. But how do people foster career adaptability, and what factors affect an individual’s development of this trait? According to career construction theory, the internal impetus of career adaptability comes from adaptive motivation, which manifests as personality and value traits as well as specific goals, personal preferences, and forms of self-recognition. In previous studies, career adaptability is positively related to proactive personality (Guan et al., 2014; Taber and Blankemeyer, 2015). Some studies show that proactive personality can predict an individual’s career adaptability (Uy et al., 2015; Hu et al., 2021). The study by Green and Batool (2017) shows that proactive personality is significantly related to all four adapting responses and supports the proposed significance of proactivity for today’s vocational environment with regard to career adaptability resources and adapting responses. This empirical research also supports that students’ proactive personality and career adaptability are a malleable and learnable construct as theorized by Savickas (2005) as well as Savickas and Porfeli (2012). Effective career education program may enhance university students’ proactivity and career adaptability resources in a sustainable manner over the long run to enable them to instinctively demonstrate career adapting responses based on the lessons learned from the intervention. Besides, some researchers think that future orientation is indicators of adaptivity and thus should relate positively to career adaptability (Rudolph et al., 2017). Studies completed by Cai et al. (2015), Guan et al. (2014), and Taber and Blankemeyer (2015) also find that future work self salience is positively correlated with career adaptability, which provides a basis for the hypothesis proposed in this study. In an increasingly complex world where work can no longer be categorized and we must spend more time learning about ourselves, future time perspective (FTP) is also an important factor in determining career adaptability. Phan et al. (2020) thinks that time, in terms of its continuity, has relevance, significance, and applicability in relation to the achievement of both educational and non-educational outcomes. They also contend that it is noteworthy for educators and researchers to develop and explore pathways, means, and/or opportunities that could encourage and foster a healthy extension of FTP (Phan et al., 2020). Several studies have demonstrated that FTP can predict career adaptability (Zacher, 2014; Jia et al., 2020). From an educational point of view, the study of future work self and time may be useful for calculating achievement, given that a student may use future orientation to guide and direct his/her academic and/or non-academic future. We believe that having a salience future work self and a deep future timespan are valuable, as this instills a sense of motivation and guides and directs a person’s cognition and behavior toward the future goals (e.g., a student’s striving to be a doctor). Therefore, this study selected proactive personality, future work self salience, and future time perspective as the embodiment of adaptive readiness in order to better understand the factors that affect career adaptability.

### Proactive Personality and Career Adaptability

According to career construction theory, career development is the product of people’s integration of their personal needs with these social expectations and, thus, their adaptation to the environment (Savickas, 2002, 2005). The career construction model of adaptation (Savickas and Porfeli, 2012; see also Tolentino et al., 2014; Hirschi et al., 2015) proposes that people’s adaptivity positively influences their career adaptability which, in turn, positively influences adapting responses and adaptation results. Possessing a subjective psychological readiness to cope with adaptation actively is an important antecedent for individuals to obtain adaptive resources and results (Savickas and Porfeli, 2012; Benmakrelouf et al., 2019). Bateman and Crant (1993) first introduced the term proactive personality and defined it as a stable psychological tendency to take actions that actively influence the surrounding environment. Initially, it was mainly used to discuss the initial component of employees in organizational behaviors. As proactive personality has gained an increasing amount of attention, however, it has been applied to other fields, such as education and career guidance. As an important manifestation of human initiative, proactive personalities can inevitably be found in Chinese culture. Some researchers think proactive personality is an important psychological resource in the college-to-career transition (e.g., Erdogan and Bauer, 2005; Brown et al., 2006). Many studies have also identified a positive relationship between proactive personality and career adaptability (Tolentino et al., 2014; Cai et al., 2015; Jiang, 2017; Hu et al., 2021) because it represents an internal motivation and behavioral tendency to spontaneously seek breakthroughs without being constrained by situational resistance. For example, Jiang (2017) finds that Proactive personality (PP) is positively related to career adaptability (CA), and PP has a stronger indirect effect on CA *via* thriving for less proactive workers. A meta-analysis of relationships with measures of adaptivity, adapting responses, and adaptation results also shows that Proactive personality belongs to adaptive readiness which relates positively to adaptability (Rudolph et al., 2017). Proactive personality can stimulate and predict career adaptability and is one of the most significant predictors of individual career adaptability (Cai et al., 2015; Hirschi et al., 2015). Based on these findings, we decide to select proactive personality as the antecedent variable in this study. Hence, on the strength of prior evidence, we formulate the following hypothesis:

*Hypothesis 1*: Proactive personality is positively related to career adaptability.

### Proactive Personality, Future Work Self Salience, and Career Adaptability

Proposed by Strauss et al. (2011), future work self is a visual representation of an individual’s future job expectations and aspirations, an internal bridge between an individual’s self-concept and future professional behavior, and the embodiment of the possible-self in the professional field (Zhang et al., 2016). Future work self includes two dimensions: future work self salience and future work self-elaboration. Zhang et al. (2016) believe that individuals constantly enrich this image through details based on clear visions of their future work to set reasonable goals and create strategies or plans for achieving goals. The development of the salience scale has allowed researchers to explore the outcome variables of future work self salience. For example, individuals with high future work self salience have more active job-hunting behaviors and higher career adaptability (Strauss et al., 2011; Guan et al., 2014). Future work self salience of new employees can also positively predict their job performance by bolstering active adaptive behavior (Zhang et al., 2014). Establishing a clear future work self is thus of great significance to career adaptability and career development and can predict the development of career adaptability. College students are typically in the exploration stage of their career development, and so they tend to be closer to future work self salience than an elaboration on the salience scale. Because of this, our study focuses on the future work self salience of college students. At the same time, existing studies have shown that proactive personality can predict future work self salience and that there is a positive correlation between these factors (Cai et al., 2015). No direct research has been conducted on whether future work self salience mediates the relationship between proactive personality and career adaptability. However, according to the preliminary judgment of the literature mentioned above, proactive personality can predict future work self salience, and future work self salience can predict career adaptability. Thus, the following hypothesis is tested as:

*Hypothesis 2*: The relationship between proactive personality and career adaptability is mediated by future work self salience.

### Future Time Perspective, Proactive Personality, and Career Adaptability

Future time perspective (FTP) is an individual’s cognitive, emotional, and behavioral tendency toward the possibility of future development. It is an ability trait as well as a dynamic trait. The future events that an individual decides to focus on as well as their future goals, plans, and behavior patterns can all be objects of concern. Future time perspective is closely related to an individual’s work value orientation, achievement motivation, and self-monitoring and has a meaningful impact on cognition, motivation, emotion, and realistic behavior. From a motivational perspective, FTP may operate to encourage a person to be purposive and self-regulated and to flourish in course of academic learning and schooling (Zimbardo and Boyd, 1999; Barber et al., 2009; Janeiro et al., 2017). In prior research, students with a high FTP tend to demonstrate strong confidence in their career decisions (Walker and Tracey, 2012; Jung et al., 2015). Some scholars show that a reciprocal relationship might exist between FTP and career adaptability, implying that career adaptability may affect FTP (Jia et al., 2020; Sb et al., 2021). While, a meta-analysis of career structure models of adaptation shows that future orientation is a psychological trait that influences career adaptation (Rudolph et al., 2017). This model views career adaptability as a resource that moderates the impact of adaptive preparation (e.g., future orientation) on adaptive outcomes (e.g., positive/negative effects; Savickas and Porfeli, 2012; Rudolph et al., 2017). Based on this argument, several studies have demonstrated that FTP can predict career adaptability (Zacher, 2014; Jia et al., 2020) and personality has also been shown to be a strong predictor of FTP (Nelson et al., 2019). Whether future time perspective mediates the relationship between proactive personality and career adaptability is a question that has not yet been directly studied. According to the preliminary judgment of the literature mentioned above, proactive personality can predict an individual’s future work self salience, while future work self salience can predict the influence of career adaptability. Thus, we hypothesize the following:

*Hypothesis 3*: The relationship between proactive personality and career adaptability is mediated by future time perspectives.

### Proactive Personality, Future Work Self Salience, Future Time Perspective, and Career Adaptability

At present, there is insufficient empirical research on whether future career self salience and future time perspective play a chain mediating role in the relationship between proactive personality and career adaptability. Proactive personality and future orientation (Bateman and Crant, 1993; Hoyle and Sherrill, 2006) are indicators of adaptivity and thus should relate positively to career adaptability. Proactive individuals successfully change their environment to better fit their needs and preferences (Bateman and Crant, 1993) and, thus, they should also be better prepared to manage career tasks and transitions than less proactive individuals. Similarly, vocational psychologists have argued that future orientation is necessary to proactively shape one’s career and to adapt to career-related challenges (Super and Knasel, 1981; Ebberwein et al., 2004). Although we believe that adaptive readiness affects adaptation, there is not much research on how this readiness affects adaptation. Time perspective is the capacity to revisit the past through our memories and to project ourselves into the future using our imagination (Savickas, 1991). We have reason to believe that a person who has a clear understanding of his future work self must have a stronger ability to project himself into the future, which will also stimulate the development of his career adaptability. Such people often show an active and positive side. As previously mentioned, proactive individuals set reasonable goals, plan, and take actions as they develop a clearer picture of their future work. These give them great insight into the future, which in turn allows them to exhibit adaptability. Thus, the following hypothesis is tested as:

*Hypothesis 4*: The relationship between proactive personality and career adaptability is mediated by the combination of future work self salience and future time perspective.

**Figure 1:**
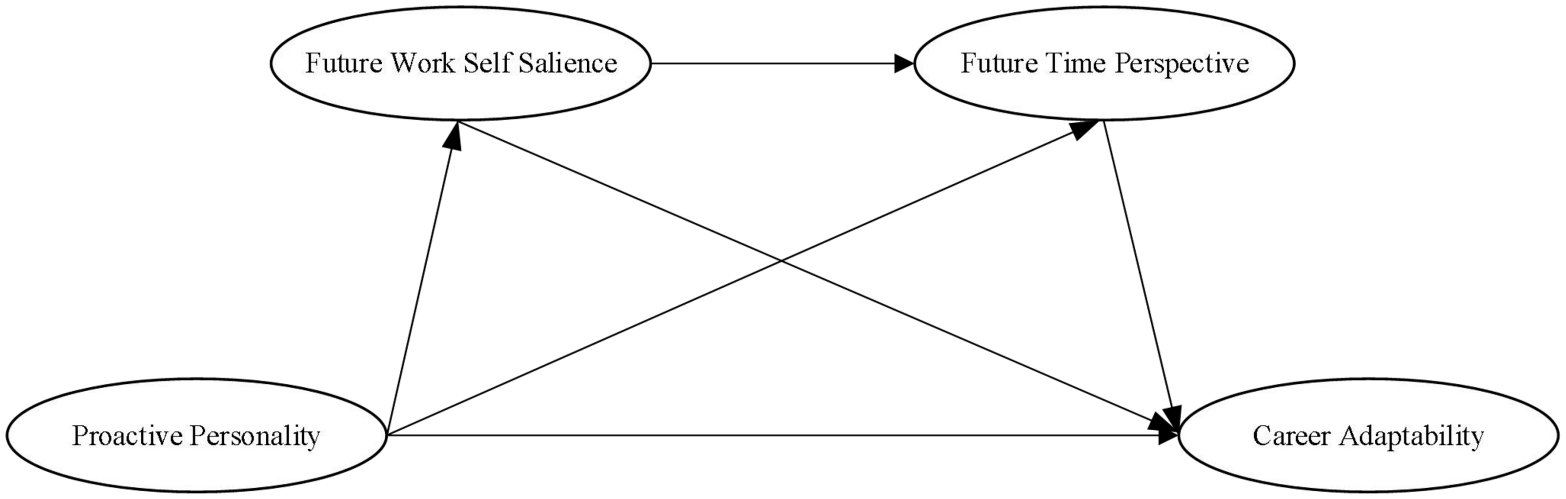
The research model.

In summary, this research explores the positive influence of proactive personality on career adaptability based on the career construction model of adaptation. We also explore the mechanisms underlying the influence of proactive personality on career adaptability, the mediating role of future work self salience, and future time perspective. In doing so, we offer implications for the career development of college students. Figure 1 depicts the study model.

## Materials and Methods

### Participants and Procedure

In this study, unified guidance language was used in the testing process. Trained psychology graduate students conducted the testing on the selected university students in China and explained the confidentiality, authenticity, and filling method of the questionnaire to the subjects. A total of 585 questionnaires were distributed to randomly selected college students from four universities in Nanjing, China. A total of 423 valid questionnaires were obtained with an effective recovery rate of 72.31%. The age of the subjects was 19.21 ± 1.14 years. The respondents consisted of 186men and 237 women. There were 121 freshmen, 101 sophomores, 92 juniors, and 109 seniors. There were 203 students majoring in humanities and social sciences, 156 students majoring in nature science, and 64 students majoring in art. A 163 of the participants were rural residents, and 260 were urban residents (see Table 1). All respondents who completed the questionnaire gave their informed consent and agreed to participate in the survey.

**Table 1:** Sample characteristics, descriptive, and frequencies.

	**Frequency**	**Valid percent (%)**
**Gender**		
Men	186	43.972
Women	237	56.028
**Grade**		
Freshmen	121	28.605
Sophomores	101	23.877
Juniors	92	21.749
Seniors	109	25.768
**Major Categories**		
Humanities and social sciences	203	47.991
Nature science	156	36.879
Art	64	15.130
**Family residence**		
Rural Residents	163	38.534
Urban Residents	260	61.466

### Methods

#### Proactive Personality

Proactive personality was measured using the brief version of the proactive personality scale, which was revised by Shang and Gan (2009) on basis of the proactive personality scale compiled by Bateman and Crant (1993). The revised PPS is a single-dimensional scale that includes 11 items. The 7-point Likert-type scoring method with a scale of 1 (totally not true) to 7 (completely true) was used in the questionnaire. Sample items of the scale are “I have been looking for a better way to do things” and “If I believe a point, there is no obstacle that can prevent me from realizing it.” All items focus on whether the individual has a positive approach to facing various difficulties. Higher scores indicate higher levels of proactive personality. In previous studies, this scale had good reliability and validity (Ma et al., 2020). In this study, the Cronbach’s alpha of the scale was 0.874.

#### Future Work Self Salience

Future work self salience was evaluated using the future work self salience scale developed by Strauss et al. (2011) based on King and Raspin’s (2004) work. The FWSS scale is a single-dimensional scale that includes 5 items. The 5-point Likert-type scoring method with a scale of 1 (totally not true) to 5 (completely true) was used in the questionnaire. Sample items of the scale are “This future is very easy for me to imagine.” All items focus on whether the individual has a future work self salience. Higher scores indicate higher levels of future work self salience. In previous studies, this scale had good reliability and validity (Gao et al., 2018a). In this study, the Cronbach’s alpha of the scale was 0.908.

#### Future Time Perspective

Future time perspective was evaluated using the future time perspective scale developed by Song (2004), which has a total of 20 topics. The scale is divided into five dimensions, including behavioral commitment, far goal orientation, future performance, purpose, and image. The 4-point Likert-type scoring method with a scale of 1 (totally not true) to 4(completely true) was used in the questionnaire. Among them, topics 2, 17, 18, 19, and 20 are reverse scoring titles. Sample items of the scale are “Once I’ve decided what to do, I think about how to get it done.” Higher scores indicate higher levels of future time perspective. In previous studies, this scale had good reliability and validity (Yang et al., 2021). In this study, the Cronbach’s alpha of the scale was 0.870.

#### Career Adaptability

Career adaptability was assessed using the career adaptability scale revised by Hou et al. (2012), which was based on the work of Savickas (2005). The 24-item scale measures career-related dimensions using four subscales: Confidence (six items; e.g., “I believe that I will insist on choosing the career I want to pursue, and try my best to practice it”), Curiosity (six items; e.g., “When confronted with career questions, I will try my best to understand them”), Concern (six items; e.g., “I will think about my future career development and direction”), and Control (six items; e.g., “I know what I want to do in the future”). The scale uses a 5-point Likert-type scoring method ranging from 1 (totally not true) to 5 (completely true). The higher the score, the higher is the individual’s career adaptability. In previous studies, this scale had good reliability and validity (Li et al., 2013). In this study, the Cronbach’s alpha of the scale was 0.958.

### Data Analysis

Common methodological bias was tested using SPSS 21.0 for Harman’s one-way test. SPSS 21.0 was also used for Pearson’s correlations. The hypothesis model was tested using the PROCESS macro in SPSS (modeling 6, 5,000 bootstrap resamples; Hayes, 2012). PROCESS macro in SPSS is a versatile computational tool for observed variable mediation, moderation, and conditional process modeling results.

### Common-Method Bias Test

Harman’s single-factor analysis was used to check for the presence of common methodological bias (Eby and Dobbins, 1997). The results indicated that there were eleven factors with eigenvalues of more than 1, in which the first one interpreted 36.826% of the variability. This result was below the threshold of 40%, which suggests that there was not a serious problem of common-method bias in this study.

### Descriptive Statistical Results

Pearson’s correlation coefficients showed significant positive correlations between proactive personality, future work self salience, future time perspective, and career adaptability (see Table 2).

**Table 2:** Means, standard deviations, and correlations.

**S. No**	**Variable**	**M**	**SD**	**1**	**2**	**3**	**4**	**5**	**6**	**7**	**8**
1.	Grade	1.560	0.497	**1**							
2.	Gender	2.450	1.157	0.260^**^	**1**						
3.	MC	1.670	0.724	−0.100^*^	0.060	**1**					
4.	FR	1.610	0.487	0.111^*^	0.016	0.144^**^	**1**				
5.	PP	5.392	0.791	−0.104^*^	0.037	0.153^**^	0.047	**1**			
6.	FTP	2.942	0.390	−0.050	0.005	0.115^*^	0.087	0.691^**^	**1**		
7.	FWSS	3.367	0.788	−0.085	0.047	0.027	0.012	0.505^**^	0.579^**^	**1**	
8.	CA	4.021	0.546	−0.004	0.013	0.045	0.062	0.711^**^	0.764^**^	0.547^**^	**1**

*N* = 423, Grade (1 = freshmen, 2 = sophomores, 3 = juniors, and 4 = seniors), Gender (1 = men; 2 = women); MC, Major Categories (1 = humanities and social sciences, 2 = nature science, and 3 = art); FR, Family Residence (1 = rural residents; 2 = urban residents); PP, Proactive Personality; FTP, Future Time Perspective; FWSS, Future Work Self Salience; CA, Career Adaptability; M, Mean; and SD, Standard Deviation. Numbers in bold on the diagonal line are Cronbach’s alpha values.

* *p* < 0.05;

** *p* < 0.01.

### Hypothesis Testing

**Figure 2:**
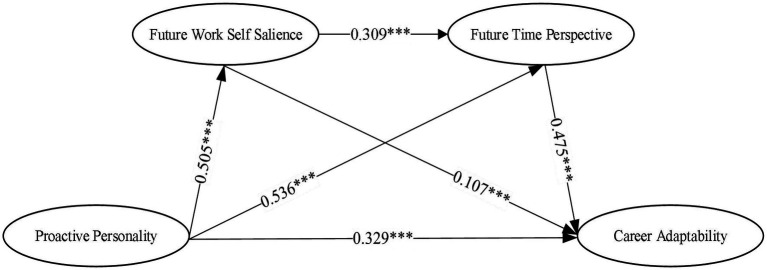
Path of the influence of proactive personality on career adaptability. ^***^p < 0.001.

Table 3 shows that proactive personality was a significant positive and valid predictor of career adaptability (*β* = 0.329, *p* < 0.001). The analysis of the mediating role of the future work self salience in proactive personality and career adaptability showed that proactive personality significantly and positively predicted future work self salience (*β* = 0.505, *p* < 0.001), and future work self salience had a significant positive effect on career adaptability (*β* = 0.107, *p* < 0.01). The analysis of the mediating role of future time perspective in proactive personality and career adaptability showed that proactive personality significantly and positively predicted the future time perspective (*β* = 0.536, *p* < 0.001), and future time perspective had a significant positive effect on career adaptability (*β* = 0.475, *p* < 0.001). The analysis of the mediating role of future work self salience and future time perspective in proactive personality and career adaptability showed that future work self salience significantly and positively predicted the future time perspective (*β* = 0.309, *p* < 0.001). So, these results supported hypotheses 1, 2, 3, and 4 (see Figure 2).

**Table 3:** The model results (*N* = 423).

**Variable**	**Model 1**			**Model 2**			**Model 3**		
**FWSS**			**FTP**			**CA**		
***β***	**SE**	*t*	***β***	**SE**	*t*	***β***	**SE**	*t*
PP	0.505	0.042	11.991^***^	0.536	0.038	14.114^***^	0.329	0.040	8.140^***^
FWSS				0.309	0.038	8.145^***^	0.107	0.036	2.980^***^
FTP							0.475	0.043	11.093^***^
*R* ^2^		0.255			0.549			0.654	
*F*		143.784^***^			255.912^***^			264.467^***^	

PP, Proactive Personality; FWSS, Future Work Self Salience; FTP, Future Time Perspective; and CA, Career Adaptability.

*** *p* < 0.001.

In these analyses, we observed the mediating effects of future work self salience and future time perspective on the relationship between proactive personality and career adaptability. Table 4 shows that the direct effect of career adaptability was 0.329. The effect size was 46.273%. The total indirect effect of future work self salience and future time perspective was 0.382. The effect size was 53.727%, which indicates a significant mediating role in the relationship between proactive personality and career adaptability. Specifically, the mediating effect consisted of the indirect effects from three pathways: (1) The mediating effect of future work self salience was 0.054 (95% CI: 0.016–0.098), and the effect size was 14.136%. (2) The mediating effect of future time perspective was 0.254(95% CI: 0.192–0.319), and the effect size was 66.492%. (3) The series of mediating effects of future work self salience and future time perspective was 0.074 (95% CI: 0.048–0.104), and the effect size is 19.372%. Both indirect effect paths were significant. Moreover, the largest mediating effect path was through proactive personality→ future time perspective→ career adaptability. The results comparing the three mediating paths suggest that proactive personality increased career adaptability primarily by increasing future work self salience and future time perspective (see Table 4).

**Table 4:** Effects and 95% confidence intervals for model.

	**Effect**	**Boot SE**	**95% Confidence Interval**	
			**LLCI**	**ULCI**
Total effect	0.711			
Direct effect	0.329	0.040	0.249	0.408
Total indirect effect	0.382	0.035	0.316	0.453
Indirect effect 1	0.054	0.021	0.016	0.098
Indirect effect 2	0.254	0.032	0.192	0.319
Indirect effect 3	0.074	0.014	0.048	0.104

Indirect effect 1 is *PP*→*FWSS*→*CA*. Indirect effect 2 is *PP*→*FTP*→*CA*. Indirect effect 3 is *PP*→*FWSS*→*FTP*→*CA*.

## Discussion

### Research Findings

This study not only verifies the significant positive predictive relationship between proactive personality and career adaptability in college students but also shows that individuals with high initiative are more willing to take actions to adapt to a career environment and achieve higher career goals (Tolentino et al., 2014) than individuals with low initiative. At the same time, it was also found that proactive personality affects the development of career adaptability through predicting factors that impact future orientation, such as future work self salience and future time perspective. Based on our findings, it can be said that proactive personality not only directly affects predictive career adaptability but also indirectly affects predictive career adaptability through future orientation factors, such as future work self salience and future time perspective. According to the theory of career construction, the development of career adaptability is affected by individual personality and subjective psychological preparation (i.e., a willingness to adapt to obtain adaptive resources and results; Savickas and Porfeli, 2012) and reflects that interaction between people and their environment can allow individuals to adapt actively. In this type of interaction, career attitude perception factors related to social cognition and focused on future exploration are particularly important for career adaptability. Future work self and boundaryless career attitudes are found to be positively correlated with individual career adaptability (Guan et al., 2014; Taber and Blankemeyer, 2015). It can be said that the discovery of such indirect effects aligns with the theoretical expectations of career construction theory.

Proactive personality→future work self salience→career adaptability mediation model results show that the more active individuals are more likely to externally explore future work and possess a clearer future work self-image. Future work self salience may enhance their confidence and control in responses to changes in the environment, which also enhances their career adaptability. Guan et al. (2014) believe that the future work self as the cognitive schema of an individual’s perception of future work reflects positive interactions with the environment and functions as an internal operation of career adaptability and intrinsic schematization of the “people – environment” role. Previous research on job seekers’ employment status has also demonstrated this effect to some extent (Taber and Blankemeyer, 2015). In a sense, the future work self is the internal bridge through which the self and environment play a role in the individual construction of the career development model. This bridge not only externalizes the self into the environment but also internalizes the environment into the self, thus promoting the process of active adaptation between the self and the environment. Proactive personality provides personality preparation for the construction of future work self and the possibility of active behavior (Gao et al., 2018b). In the process of promoting the continued salience of the future work self, proactive personality provides adaptive motivation for the improvement of career adaptability.

The results of the mediating model of proactive personality→future time perspective→career adaptability suggest that proactive individuals have greater future time perspective and exhibit stronger career adaptability during career development. As a mediating variable between proactive personality and career adaptability, the future time perspective provides a long-term perspective for the impact of proactive personality on career adaptability and may help individuals set incentive goals and develop long-term behaviors to achieve these objectives (Vansteenkiste et al., 2004; Lin et al., 2014). Proactive personality encourages individuals to prepare for long-term behaviors and long-term motivation goals. It not only encourages individuals to take initiative but also makes them more resilient and strengthens their will. The improvement of future time perspective makes individuals think that the future is infinitely open and that time is abundant, and they are more likely to choose targets that can prepare them for the future, expand their horizons, and allow them to socialize extensively and expand their social circle (Carstensen, 2006; Maki et al., 2016). Therefore, high future time perspective may have two effects. First, it could allow an individual to make more friends and broaden their social circle. Second, it could allow them to acquire more information that allows them to prepare for the future. Career adaptability, on the other hand, develops in the context of possible longer-range motivation goals and behaviors (Najib and Aljanabi, 2020), which could allow individuals to become more resilient rather than blindly adapting for the sake of it.

The results of the mediating model of proactive personality→ the future work self salience→ future time perspective→ career adaptability show that proactive personality individuals have a strong future time perspective and a clearer future work self-cognitive schemata because they embrace initiative exploration, which allows them to take lasting actions that affect their ability to meet incentive targets. Career adaptability is thus linked to stronger vitality, tenacity, and an ability to cope with changing environments. For college students in early adulthood who are in the exploratory stage of career development, proactive personality characteristics help them actively explore and promote their formation of a clear future work self. This clear representation of future work can guide and support their pursuit of goals and long-term behaviors. Individuals can make career progress by actively seeking breakthroughs (Uy et al., 2015) and integrating past experience with current perception and future planning, and in the process, they often improve their career adaptability.

The results of this study show that the influence of adaptive readiness factors (such as proactive personality, future work self salience, and future time perspective) on career adaptability are not simply juxtaposed but have an internal structure. It may be that more proactive people are more likely to develop a clear future working self. Such clear self-cognition and working imagination make them better equipped to deal with the future, more purposeful and planned. Thus, it provides personality, imagination, and timely preparation for career adaptability. These findings not only supplement the theory of career construction but also have important practical guiding significance on how to carry out career education among college students.

### Practical Implications

In practice, procrastination hurts college students because it often manifests as powerlessness. Some research finds that procrastination is one of the main studying influence factors which result in students delaying learning, obtaining academic debts, and being exposed to dropout risk before reaching their career goals (Katane and Jerkunkova, 2020). Procrastination is not uncommon among Chinese college students, some researchers even found that Chinese students scored higher on procrastination than American college students (Yan et al., 2015). Students are unable to plan their own time and efficiently self-manage their activities in today’s higher education environment, which increases their risk of dropping out of school (Katane and Jerkunkova, 2020). Slow employment and no employment have become normalized, and many college students lack the desire to explore future careers. Their vision for the future is blank, and they lack the guidance that they need to construct long-term career goals. Because of this, they are slow to develop their career initiative, which affects their quality of employment after graduation. The results of this study show that proactive personality, future work self salience, and future time perspective have positive and predictive effects on college students’ career adaptability. This finding has practical significance for career development in university because it shows that if college students possess the qualities of desire and active inquiry, their imagination and clarity about their future work self will grow, which will, in turn, allow them to develop the motivation they need to construct long-term goals and strengthen their career adaptability. Consequently, career education should work to foster initiative, desire, self-exploration, and long-term motivational goals in an effort to help students construct clear future work self-images. Career adaptability is an important prerequisite for achieving a person-job fit in frequent cross-border activities and unstable employment environments (Guan et al., 2021) and should thus be emphasized in career education initiatives that take place at the college level.

How to help college students prepare for adaptation? First, we should establish a whole-process, stratified, and focused career education system for college students. We can set up targeted career education courses for students of different grades. For example, career awareness courses are offered for freshmen to help them sort out their career goals and learn about college planning. Career ability improvement courses are offered for sophomores and juniors to provide their ability and technical support for their subsequent employment or further study. In graduation grade, they will be provided with specialized employment guidance courses to help them better cope with employment. Second, give students a sense of purpose for their career and learn to break down goals. At present, career education courses in many universities in China are constructed around the concept of person-job. We think we should break the usual teaching philosophy, pay more attention to students’ own career development goals, and return to their study and life (see Table 5). Career education having a high affective value is one that is current, relevant, practical, and thought-provoking to motivate learners to integrate the lessons learned from the training into their daily lives (Green and Batool, 2017). Career education courses are designed around self-imagination, long-term goals, and short-term goals of future work, and more exploratory activities and practices are added to the courses to help them explore actively, motivate them to take more actions, drive their imagination, and stimulate their initiative through actions.

**Table 5:** Curriculum adjustment of college career education.

**S. No.**	**Original course modules**	**Revised course modules**
1.	Introduction: career awareness	My university: my school and my study
2.	Interest exploration	What do I have: my current situation
3.	Character exploration	What else can I do (I): work world exploration (in class)
4.	Skill exploration	What else can I do (II): work world exploration (extracurricular)
5.	Values exploration	What else can I do (III): discovery of exploration
6.	Work world exploration (I)	What else can I do (IV): ability construction
7.	Work world exploration (II)	What else can I do (V): goal setting and implementation
8.	Decision making and action plan	My university: the switch between disorder and order

## Limitations and Future Research

As a form of horizontal research, this study essentially did not reveal the causal relationship between variables. Given the limited generalizability of the current research results, future replicative studies that take place at different times and between different groups could further verify the relationship between proactive personality, future work self, future time perspective, and career adaptability. Some quasi-experimental designs or qualitative research can also be used to further explore the relationship between these variables. Many factors influence career adaptability. This study only focused on some of those variables (i.e., proactive personality, future work self salience, and future time perspective) and did not establish the overall network of influencing factors of career adaptability. In the future, influencing factors of career adaptability can be further discussed from other perspectives, such as individual coping strategies, career development situations, and population culture, as a means of establishing the overall promotion strategy of career adaptability.

## Conclusion

In conclusion, this study once again verifies that proactive personality, future work self salience, and future time perspective have positively predictive effects on the career adaptability of Chinese adolescents. The proactive personality of adolescents affects their future work self salience, which influences their future time perspective and career adaptability.

## Data Availability Statement

The original contributions presented in the study are included in the article/supplementary material, further inquiries can be directed to the corresponding author.

## Ethics Statement

This study involving human participants was reviewed and approved by the Ethics Committee of Nanjing Normal University. The Ethics Committee waived the requirement of written informed consent for participation.

## Author Contributions

HL put forward the core point of this research and wrote the paper. JW and ST participated in writing and data analysis. XL and JW were responsible for data collection and modifying the manuscript. XG and XL supervised the topic selection and research design. All authors contributed to the article and approved the submitted version.

## Funding

This study was funded by the Jiangsu Province University’s Advantageous Discipline Construction Project, grant number PAPD.

## Conflict of Interest

The authors declare that the research was conducted in the absence of any commercial or financial relationships that could be construed as a potential conflict of interest.

## Publisher’s Note

All claims expressed in this article are solely those of the authors and do not necessarily represent those of their affiliated organizations, or those of the publisher, the editors and the reviewers. Any product that may be evaluated in this article, or claim that may be made by its manufacturer, is not guaranteed or endorsed by the publisher.
